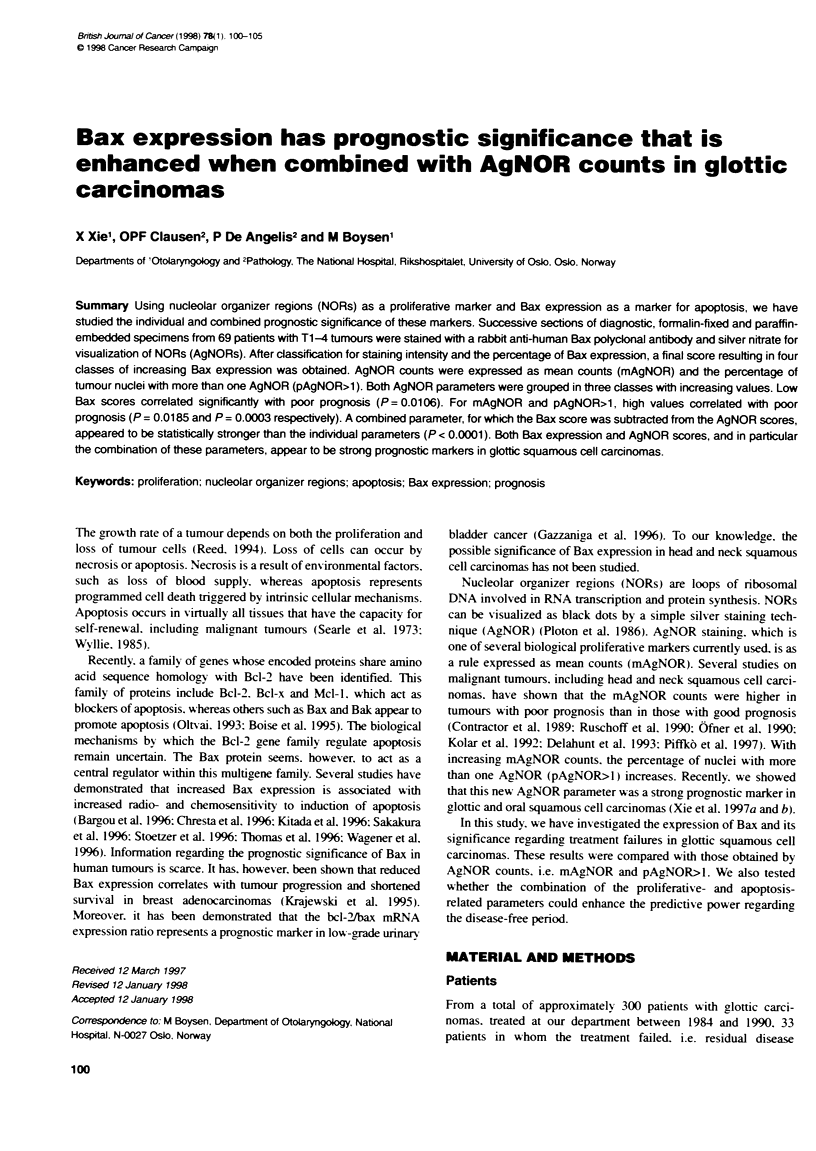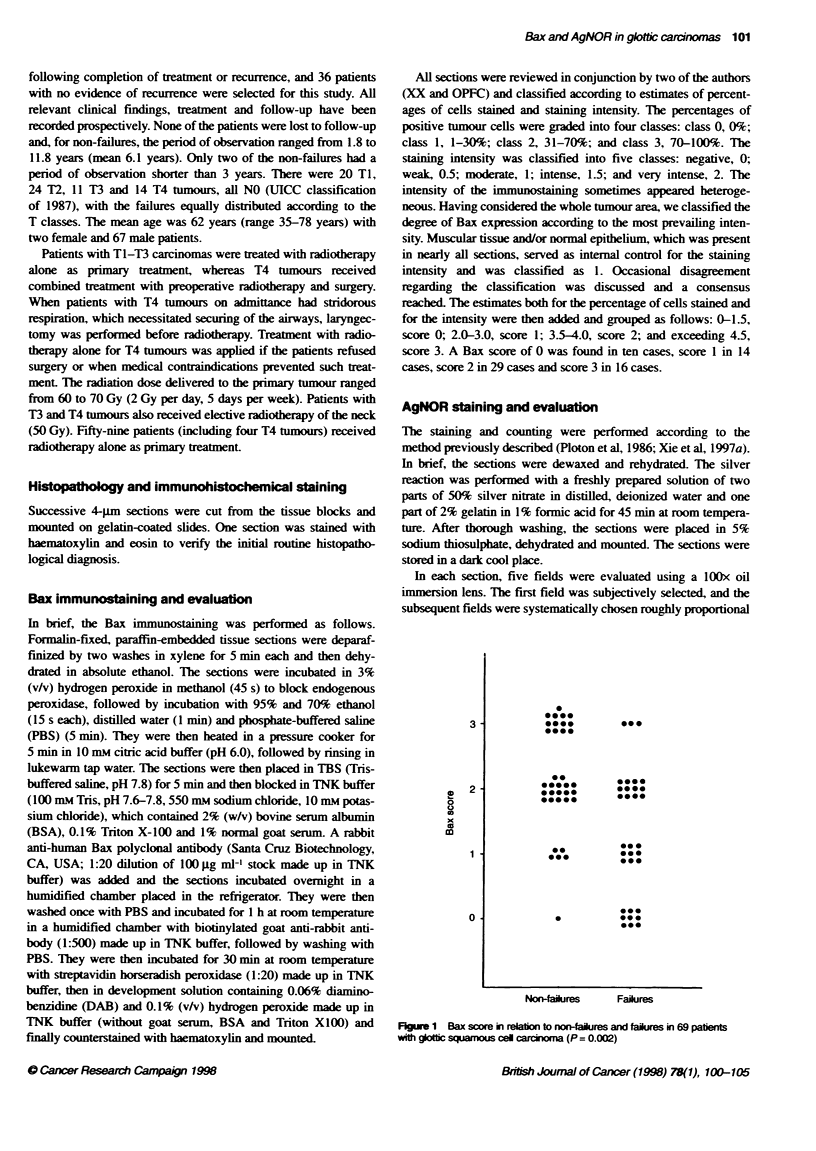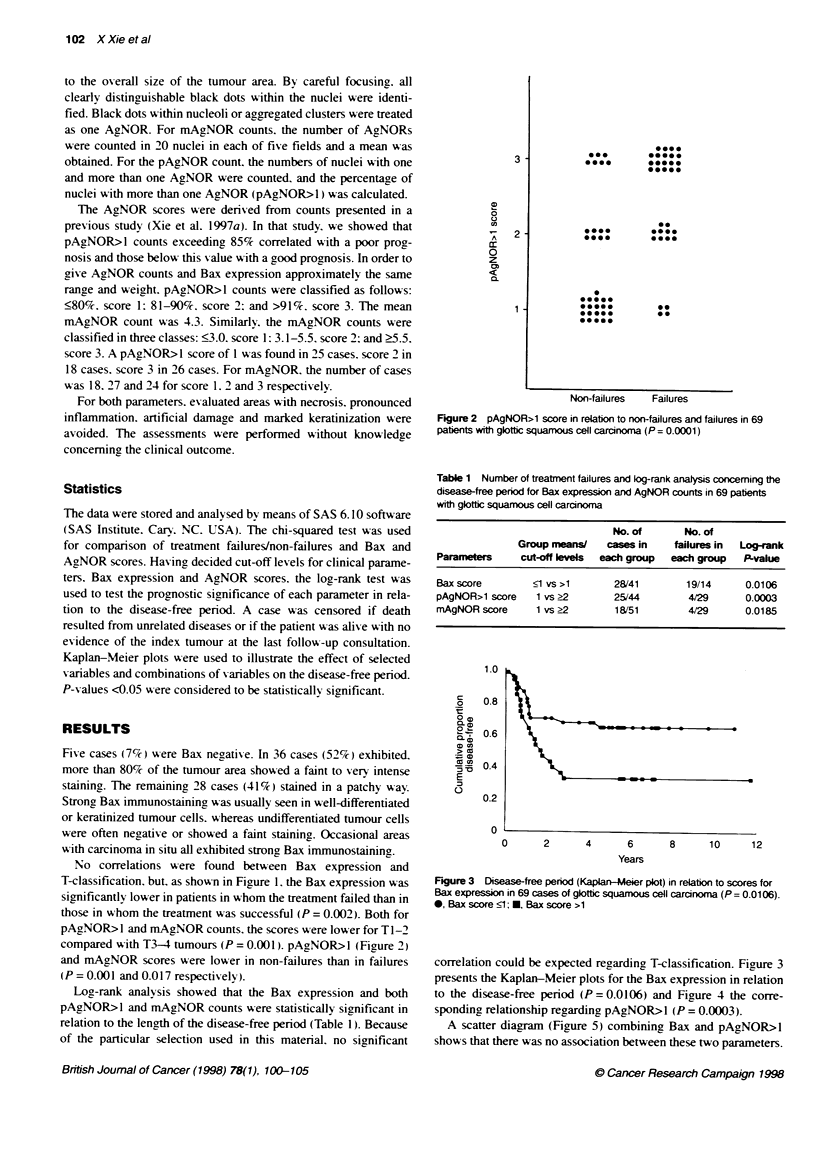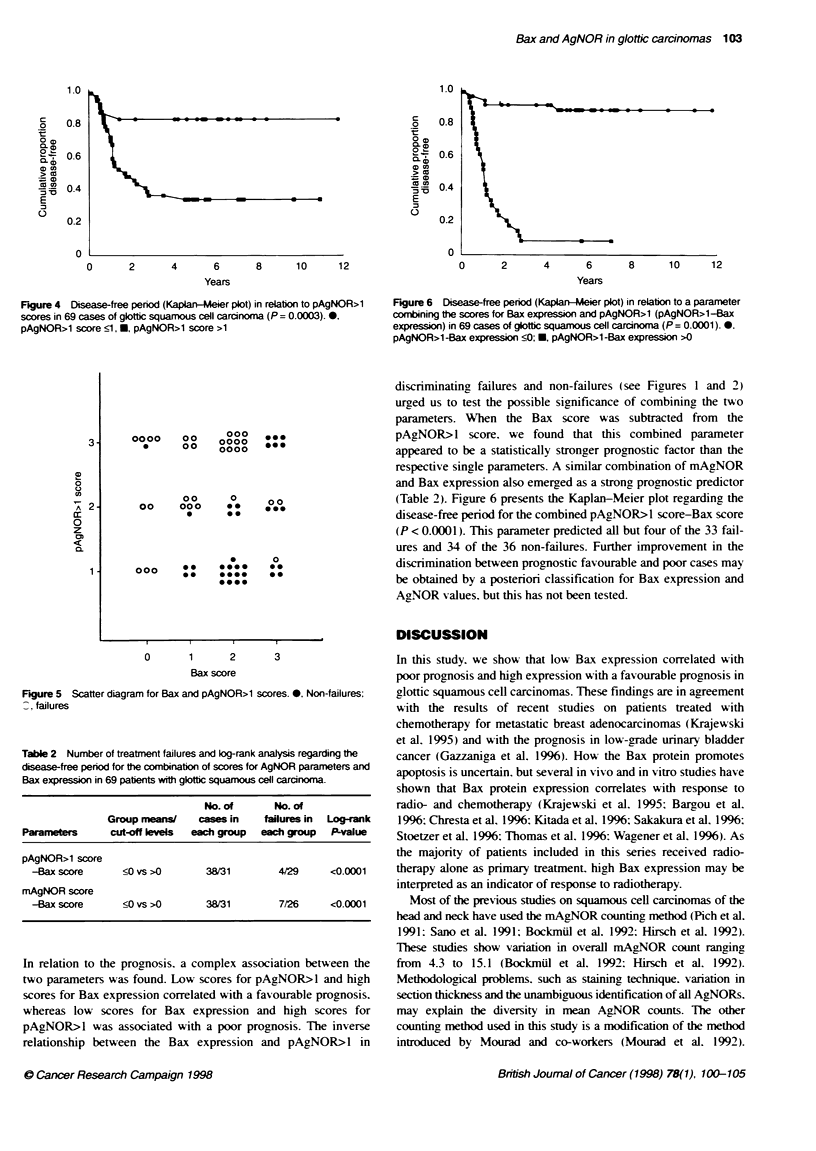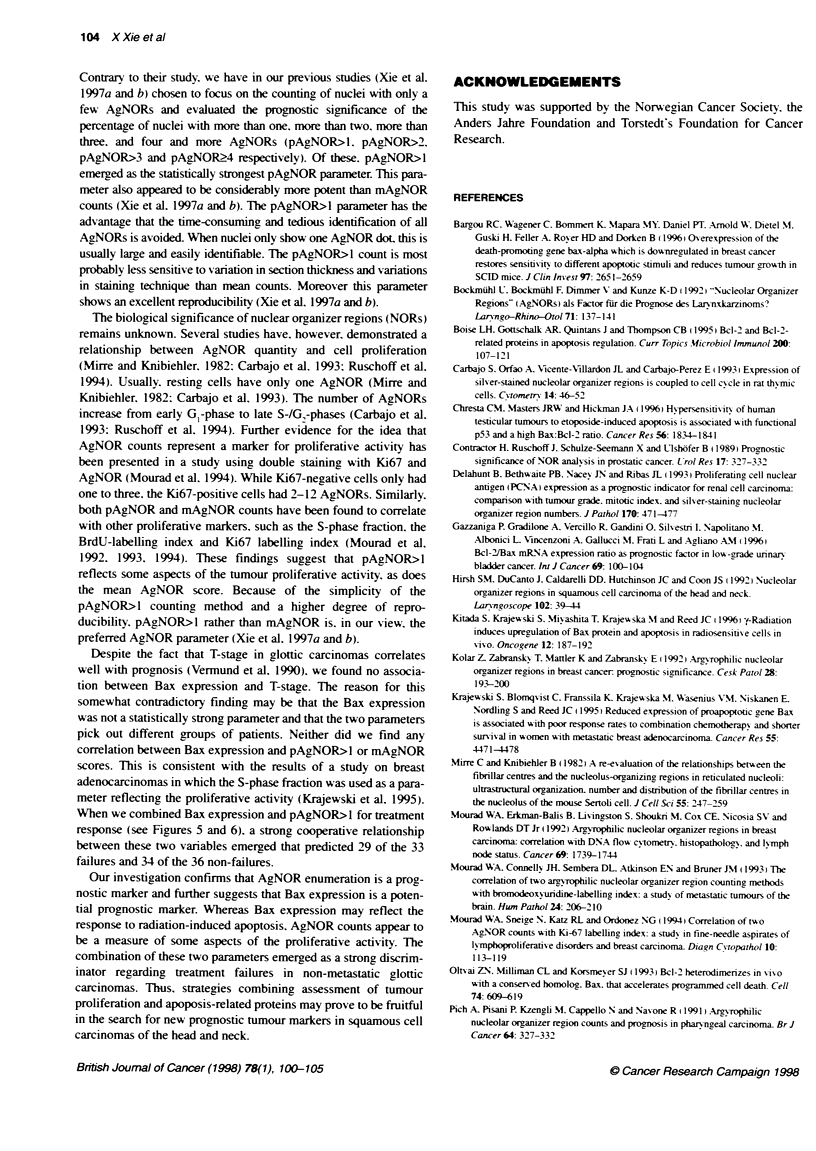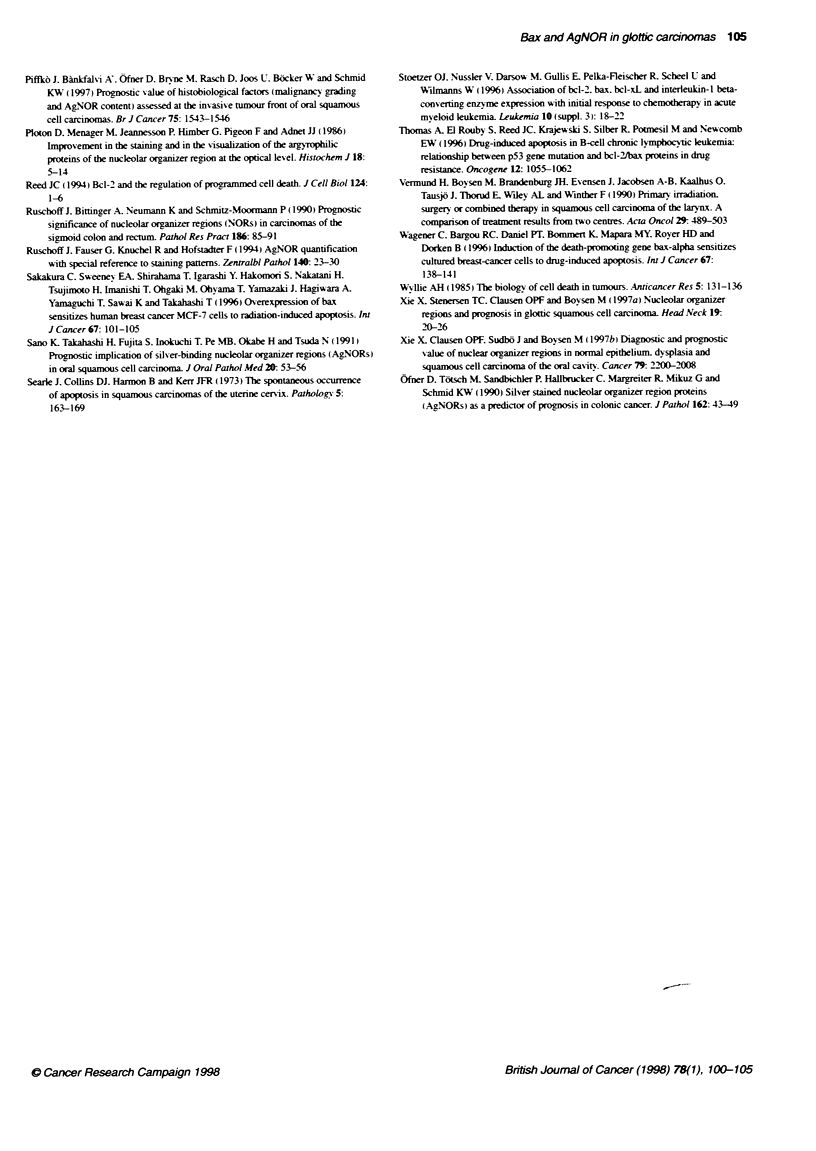# Bax expression has prognostic significance that is enhanced when combined with AgNOR counts in glottic carcinomas.

**DOI:** 10.1038/bjc.1998.449

**Published:** 1998-07

**Authors:** X. Xie, O. P. Clausen, P. De Angelis, M. Boysen

**Affiliations:** Department of Otolaryngology, The National Hospital, Rikshospitalet, University of Oslo, Norway.

## Abstract

Using nucleolar organizer regions (NORs) as a proliferative marker and Bax expression as a marker for apoptosis, we have studied the individual and combined prognostic significance of these markers. Successive sections of diagnostic, formalin-fixed and paraffin-embedded specimens from 69 patients with T1-4 tumours were stained with a rabbit anti-human Bax polyclonal antibody and silver nitrate for visualization of NORs (AgNORs). After classification for staining intensity and the percentage of Bax expression, a final score resulting in four classes of increasing Bax expression was obtained. AgNOR counts were expressed as mean counts (mAgNOR) and the percentage of tumour nuclei with more than one AgNOR (pAgNOR>1). Both AgNOR parameters were grouped in three classes with increasing values. Low Bax scores correlated significantly with poor prognosis (P = 0.0106). For mAgNOR and pAgNOR>1, high values correlated with poor prognosis (P = 0.0185 and P = 0.0003 respectively). A combined parameter, for which the Bax score was subtracted from the AgNOR scores, appeared to be statistically stronger than the individual parameters (P < 0.0001). Both Bax expression and AgNOR scores, and in particular the combination of these parameters, appear to be strong prognostic markers in glottic squamous cell carcinomas.


British Journal of Cancer (1998) 78(1). 100–105
© 1998 Cancer Research Campaign

### Bax expression has prognostic significance that is enhanced when combined with AgNOR counts in glottic carcinomas

**X Xie[1], OPF Clausen[2], P De Angelis[2] and M Boysen[1]**

Departments of [1]Otolaryngology and [2]Pathology, The National Hospital, Rikshospitalet, University of Oslo, Oslo, Norway

**Summary** Using nucleolar organizer regions (NORs) as a proliferative marker and Bax expression as a marker for apoptosis, we have studied the individual and combined prognostic significance of these markers. Successive sections of diagnostic, formalin-fixed and paraffin-embedded specimens from 69 patients with T1–4 tumours were stained with a rabbit anti-human Bax polyclonal antibody and silver nitrate for visualization of NORs (AgNORs). After classification for staining intensity and the percentage of Bax expression, a final score resulting in four classes of increasing Bax expression was obtained. AgNOR counts were expressed as mean counts (mAgNOR) and the percentage of tumour nuclei with more than one AgNOR (pAgNOR>1). Both AgNOR parameters were grouped in three classes with increasing values. Low Bax scores correlated significantly with poor prognosis ($P = 0.0106$). For mAgNOR and pAgNOR>1, high values correlated with poor prognosis ($P = 0.0185$ and $P = 0.0003$ respectively). A combined parameter, for which the Bax score was subtracted from the AgNOR scores, appeared to be statistically stronger than the individual parameters ($P < 0.0001$). Both Bax expression and AgNOR scores, and in particular the combination of these parameters, appear to be strong prognostic markers in glottic squamous cell carcinomas.

**Keywords:** proliferation; nucleolar organizer regions; apoptosis; Bax expression; prognosis

The growth rate of a tumour depends on both the proliferation and loss of tumour cells (Reed, 1994). Loss of cells can occur by necrosis or apoptosis. Necrosis is a result of environmental factors, such as loss of blood supply, whereas apoptosis represents programmed cell death triggered by intrinsic cellular mechanisms. Apoptosis occurs in virtually all tissues that have the capacity for self-renewal, including malignant tumours (Searle et al, 1973; Wyllie, 1985).

Recently, a family of genes whose encoded proteins share amino acid sequence homology with Bcl-2 have been identified. This family of proteins include Bcl-2, Bcl-x and Mcl-1, which act as blockers of apoptosis, whereas others such as Bax and Bak appear to promote apoptosis (Oltvai, 1993; Boise et al, 1995). The biological mechanisms by which the Bcl-2 gene family regulate apoptosis remain uncertain. The Bax protein seems, however, to act as a central regulator within this multigene family. Several studies have demonstrated that increased Bax expression is associated with increased radio- and chemosensitivity to induction of apoptosis (Bargou et al, 1996; Chresta et al, 1996; Kitada et al, 1996; Sakakura et al, 1996; Stoetzer et al, 1996; Thomas et al, 1996; Wagener et al, 1996). Information regarding the prognostic significance of Bax in human tumours is scarce. It has, however, been shown that reduced Bax expression correlates with tumour progression and shortened survival in breast adenocarcinomas (Krajewski et al, 1995). Moreover, it has been demonstrated that the bcl-2/bax mRNA expression ratio represents a prognostic marker in low-grade urinary bladder cancer (Gazzaniga et al, 1996). To our knowledge, the possible significance of Bax expression in head and neck squamous cell carcinomas has not been studied.

Nucleolar organizer regions (NORs) are loops of ribosomal DNA involved in RNA transcription and protein synthesis. NORs can be visualized as black dots by a simple silver staining technique (AgNOR) (Ploton et al, 1986). AgNOR staining, which is one of several biological proliferative markers currently used, is as a rule expressed as mean counts (mAgNOR). Several studies on malignant tumours, including head and neck squamous cell carcinomas, have shown that the mAgNOR counts were higher in tumours with poor prognosis than in those with good prognosis (Contractor et al, 1989; Ruschoff et al, 1990; Öfner et al, 1990; Kolar et al, 1992; Delahunt et al, 1993; Piffkò et al, 1997). With increasing mAgNOR counts, the percentage of nuclei with more than one AgNOR (pAgNOR>1) increases. Recently, we showed that this new AgNOR parameter was a strong prognostic marker in glottic and oral squamous cell carcinomas (Xie et al, 1997*a* and *b*).

In this study, we have investigated the expression of Bax and its significance regarding treatment failures in glottic squamous cell carcinomas. These results were compared with those obtained by AgNOR counts, i.e. mAgNOR and pAgNOR>1. We also tested whether the combination of the proliferative- and apoptosis-related parameters could enhance the predictive power regarding the disease-free period.

*Received 12 March 1997*
*Revised 12 January 1998*
*Accepted 12 January 1998*

*Correspondence to:* M Boysen, Department of Otolaryngology, National Hospital, N-0027 Oslo, Norway

### MATERIAL AND METHODS

### Patients

From a total of approximately 300 patients with glottic carcinomas, treated at our department between 1984 and 1990, 33 patients in whom the treatment failed, i.e. residual disease following completion of treatment or recurrence, and 36 patients with no evidence of recurrence were selected for this study. All relevant clinical findings, treatment and follow-up have been recorded prospectively. None of the patients were lost to follow-up and, for non-failures, the period of observation ranged from 1.8 to 11.8 years (mean 6.1 years). Only two of the non-failures had a period of observation shorter than 3 years. There were 20 T1, 24 T2, 11 T3 and 14 T4 tumours, all N0 (UICC classification of 1987), with the failures equally distributed according to the T classes. The mean age was 62 years (range 35–78 years) with two female and 67 male patients.

Patients with T1–T3 carcinomas were treated with radiotherapy alone as primary treatment, whereas T4 tumours received combined treatment with preoperative radiotherapy and surgery. When patients with T4 tumours on admittance had stridorous respiration, which necessitated securing of the airways, laryngectomy was performed before radiotherapy. Treatment with radiotherapy alone for T4 tumours was applied if the patients refused surgery or when medical contraindications prevented such treatment. The radiation dose delivered to the primary tumour ranged from 60 to 70 Gy (2 Gy per day, 5 days per week). Patients with T3 and T4 tumours also received elective radiotherapy of the neck (50 Gy). Fifty-nine patients (including four T4 tumours) received radiotherapy alone as primary treatment.

### Histopathology and immunohistochemical staining

Successive 4-μm sections were cut from the tissue blocks and mounted on gelatin-coated slides. One section was stained with haematoxylin and eosin to verify the initial routine histopathological diagnosis.

### Bax immunostaining and evaluation

In brief, the Bax immunostaining was performed as follows. Formalin-fixed, paraffin-embedded tissue sections were deparaffinized by two washes in xylene for 5 min each and then dehydrated in absolute ethanol. The sections were incubated in 3% (v/v) hydrogen peroxide in methanol (45 s) to block endogenous peroxidase, followed by incubation with 95% and 70% ethanol (15 s each), distilled water (1 min) and phosphate-buffered saline (PBS) (5 min). They were then heated in a pressure cooker for 5 min in 10 mM citric acid buffer (pH 6.0), followed by rinsing in lukewarm tap water. The sections were then placed in TBS (Tris-buffered saline, pH 7.8) for 5 min and then blocked in TNK buffer (100 mM Tris, pH 7.6–7.8, 550 mM sodium chloride, 10 mM potassium chloride), which contained 2% (w/v) bovine serum albumin (BSA), 0.1% Triton X-100 and 1% normal goat serum. A rabbit anti-human Bax polyclonal antibody (Santa Cruz Biotechnology, CA, USA; 1:20 dilution of 100 μg $ml^{-1}$ stock made up in TNK buffer) was added and the sections incubated overnight in a humidified chamber placed in the refrigerator. They were then washed once with PBS and incubated for 1 h at room temperature in a humidified chamber with biotinylated goat anti-rabbit antibody (1:500) made up in TNK buffer, followed by washing with PBS. They were then incubated for 30 min at room temperature with streptavidin horseradish peroxidase (1:20) made up in TNK buffer, then in development solution containing 0.06% diaminobenzidine (DAB) and 0.1% (v/v) hydrogen peroxide made up in TNK buffer (without goat serum, BSA and Triton X100) and finally counterstained with haematoxylin and mounted.

All sections were reviewed in conjunction by two of the authors (XX and OPFC) and classified according to estimates of percentages of cells stained and staining intensity. The percentages of positive tumour cells were graded into four classes: class 0, 0%; class 1, 1–30%; class 2, 31–70%; and class 3, 70–100%. The staining intensity was classified into five classes: negative, 0; weak, 0.5; moderate, 1; intense, 1.5; and very intense, 2. The intensity of the immunostaining sometimes appeared heterogeneous. Having considered the whole tumour area, we classified the degree of Bax expression according to the most prevailing intensity. Muscular tissue and/or normal epithelium, which was present in nearly all sections, served as internal control for the staining intensity and was classified as 1. Occasional disagreement regarding the classification was discussed and a consensus reached. The estimates both for the percentage of cells stained and for the intensity were then added and grouped as follows: 0–1.5, score 0; 2.0–3.0, score 1; 3.5–4.0, score 2; and exceeding 4.5, score 3. A Bax score of 0 was found in ten cases, score 1 in 14 cases, score 2 in 29 cases and score 3 in 16 cases.

### AgNOR staining and evaluation

The staining and counting were performed according to the method previously described (Ploton et al, 1986; Xie et al, 1997*a*). In brief, the sections were dewaxed and rehydrated. The silver reaction was performed with a freshly prepared solution of two parts of 50% silver nitrate in distilled, deionized water and one part of 2% gelatin in 1% formic acid for 45 min at room temperature. After thorough washing, the sections were placed in 5% sodium thiosulphate, dehydrated and mounted. The sections were stored in a dark cool place.

In each section, five fields were evaluated using a 100× oil immersion lens. The first field was subjectively selected, and the subsequent fields were systematically chosen roughly proportional

**Figure 1** Bax score in relation to non-failures and failures in 69 patients with glottic squamous cell carcinoma ($P = 0.002$)

to the overall size of the tumour area. By careful focusing, all clearly distinguishable black dots within the nuclei were identified. Black dots within nucleoli or aggregated clusters were treated as one AgNOR. For mAgNOR counts, the number of AgNORs were counted in 20 nuclei in each of five fields and a mean was obtained. For the pAgNOR count, the numbers of nuclei with one and more than one AgNOR were counted, and the percentage of nuclei with more than one AgNOR (pAgNOR>1) was calculated.

The AgNOR scores were derived from counts presented in a previous study (Xie et al, 1997*a*). In that study, we showed that pAgNOR>1 counts exceeding 85% correlated with a poor prognosis and those below this value with a good prognosis. In order to give AgNOR counts and Bax expression approximately the same range and weight, pAgNOR>1 counts were classified as follows: ≤80%, score 1; 81–90%, score 2; and >91%, score 3. The mean mAgNOR count was 4.3. Similarly, the mAgNOR counts were classified in three classes: ≤3.0, score 1; 3.1–5.5, score 2; and ≥5.5, score 3. A pAgNOR>1 score of 1 was found in 25 cases, score 2 in 18 cases, score 3 in 26 cases. For mAgNOR, the number of cases was 18, 27 and 24 for score 1, 2 and 3 respectively.

For both parameters, evaluated areas with necrosis, pronounced inflammation, artificial damage and marked keratinization were avoided. The assessments were performed without knowledge concerning the clinical outcome.

### Statistics

The data were stored and analysed by means of SAS 6.10 software (SAS Institute, Cary, NC, USA). The chi-squared test was used for comparison of treatment failures/non-failures and Bax and AgNOR scores. Having decided cut-off levels for clinical parameters, Bax expression and AgNOR scores, the log-rank test was used to test the prognostic significance of each parameter in relation to the disease-free period. A case was censored if death resulted from unrelated diseases or if the patient was alive with no evidence of the index tumour at the last follow-up consultation. Kaplan–Meier plots were used to illustrate the effect of selected variables and combinations of variables on the disease-free period. *P*-values <0.05 were considered to be statistically significant.

### RESULTS

Five cases (7%) were Bax negative. In 36 cases (52%) exhibited, more than 80% of the tumour area showed a faint to very intense staining. The remaining 28 cases (41%) stained in a patchy way. Strong Bax immunostaining was usually seen in well-differentiated or keratinized tumour cells, whereas undifferentiated tumour cells were often negative or showed a faint staining. Occasional areas with carcinoma in situ all exhibited strong Bax immunostaining.

No correlations were found between Bax expression and T-classification, but, as shown in Figure 1, the Bax expression was significantly lower in patients in whom the treatment failed than in those in whom the treatment was successful ($P = 0.002$). Both for pAgNOR>1 and mAgNOR counts, the scores were lower for T1–2 compared with T3–4 tumours ($P = 0.001$). pAgNOR>1 (Figure 2) and mAgNOR scores were lower in non-failures than in failures ($P = 0.001$ and 0.017 respectively).

Log-rank analysis showed that the Bax expression and both pAgNOR>1 and mAgNOR counts were statistically significant in relation to the length of the disease-free period (Table 1). Because of the particular selection used in this material, no significant correlation could be expected regarding T-classification. Figure 3 presents the Kaplan–Meier plots for the Bax expression in relation to the disease-free period ($P = 0.0106$) and Figure 4 the corresponding relationship regarding pAgNOR>1 ($P = 0.0003$).

A scatter diagram (Figure 5) combining Bax and pAgNOR>1 shows that there was no association between these two parameters.

**Figure 2** pAgNOR>1 score in relation to non-failures and failures in 69 patients with glottic squamous cell carcinoma ($P = 0.0001$)

**Table 1** Number of treatment failures and log-rank analysis concerning the disease-free period for Bax expression and AgNOR counts in 69 patients with glottic squamous cell carcinoma

| Parameters | Group means/ cut-off levels | No. of cases in each group | No. of failures in each group | Log-rank *P*-value |
|---|---|---|---|---|
| Bax score | ≤1 vs >1 | 28/41 | 19/14 | 0.0106 |
| pAgNOR>1 score | 1 vs ≥2 | 25/44 | 4/29 | 0.0003 |
| mAgNOR score | 1 vs ≥2 | 18/51 | 4/29 | 0.0185 |

**Figure 3** Disease-free period (Kaplan–Meier plot) in relation to scores for Bax expression in 69 cases of glottic squamous cell carcinoma ($P = 0.0106$). ●, Bax score ≤1; ■, Bax score >1

© Cancer Research Campaign 1998

**Figure 4** Disease-free period (Kaplan–Meier plot) in relation to pAgNOR>1 scores in 69 cases of glottic squamous cell carcinoma ($P = 0.0003$). ●, pAgNOR>1 score ≤1, ■, pAgNOR>1 score >1

**Figure 6** Disease-free period (Kaplan–Meier plot) in relation to a parameter combining the scores for Bax expression and pAgNOR>1 (pAgNOR>1–Bax expression) in 69 cases of glottic squamous cell carcinoma ($P = 0.0001$). ●, pAgNOR>1-Bax expression ≤0; ■, pAgNOR>1-Bax expression >0

**Figure 5** Scatter diagram for Bax and pAgNOR>1 scores. ●, Non-failures; ○, failures

**Table 2** Number of treatment failures and log-rank analysis regarding the disease-free period for the combination of scores for AgNOR parameters and Bax expression in 69 patients with glottic squamous cell carcinoma.

| Parameters | Group means/ cut-off levels | No. of cases in each group | No. of failures in each group | Log-rank *P*-value |
|---|---|---|---|---|
| pAgNOR>1 score –Bax score | ≤0 vs >0 | 38/31 | 4/29 | <0.0001 |
| mAgNOR score –Bax score | ≤0 vs >0 | 38/31 | 7/26 | <0.0001 |

In relation to the prognosis, a complex association between the two parameters was found. Low scores for pAgNOR>1 and high scores for Bax expression correlated with a favourable prognosis, whereas low scores for Bax expression and high scores for pAgNOR>1 was associated with a poor prognosis. The inverse relationship between the Bax expression and pAgNOR>1 in discriminating failures and non-failures (see Figures 1 and 2) urged us to test the possible significance of combining the two parameters. When the Bax score was subtracted from the pAgNOR>1 score, we found that this combined parameter appeared to be a statistically stronger prognostic factor than the respective single parameters. A similar combination of mAgNOR and Bax expression also emerged as a strong prognostic predictor (Table 2). Figure 6 presents the Kaplan–Meier plot regarding the disease-free period for the combined pAgNOR>1 score–Bax score ($P < 0.0001$). This parameter predicted all but four of the 33 failures and 34 of the 36 non-failures. Further improvement in the discrimination between prognostic favourable and poor cases may be obtained by a posteriori classification for Bax expression and AgNOR values, but this has not been tested.

### DISCUSSION

In this study, we show that low Bax expression correlated with poor prognosis and high expression with a favourable prognosis in glottic squamous cell carcinomas. These findings are in agreement with the results of recent studies on patients treated with chemotherapy for metastatic breast adenocarcinomas (Krajewski et al, 1995) and with the prognosis in low-grade urinary bladder cancer (Gazzaniga et al, 1996). How the Bax protein promotes apoptosis is uncertain, but several in vivo and in vitro studies have shown that Bax protein expression correlates with response to radio- and chemotherapy (Krajewski et al, 1995; Bargou et al, 1996; Chresta et al, 1996; Kitada et al, 1996; Sakakura et al, 1996; Stoetzer et al, 1996; Thomas et al, 1996; Wagener et al, 1996). As the majority of patients included in this series received radiotherapy alone as primary treatment, high Bax expression may be interpreted as an indicator of response to radiotherapy.

Most of the previous studies on squamous cell carcinomas of the head and neck have used the mAgNOR counting method (Pich et al, 1991; Sano et al, 1991; Bockmül et al, 1992; Hirsch et al, 1992). These studies show variation in overall mAgNOR count ranging from 4.3 to 15.1 (Bockmül et al, 1992; Hirsch et al, 1992). Methodological problems, such as staining technique, variation in section thickness and the unambiguous identification of all AgNORs, may explain the diversity in mean AgNOR counts. The other counting method used in this study is a modification of the method introduced by Mourad and co-workers (Mourad et al, 1992).

Contrary to their study, we have in our previous studies (Xie et al. 1997*a* and *b*) chosen to focus on the counting of nuclei with only a few AgNORs and evaluated the prognostic significance of the percentage of nuclei with more than one, more than two, more than three, and four and more AgNORs (pAgNOR>1, pAgNOR>2, pAgNOR>3 and pAgNOR≥4 respectively). Of these, pAgNOR>1 emerged as the statistically strongest pAgNOR parameter. This parameter also appeared to be considerably more potent than mAgNOR counts (Xie et al. 1997*a* and *b*). The pAgNOR>1 parameter has the advantage that the time-consuming and tedious identification of all AgNORs is avoided. When nuclei only show one AgNOR dot, this is usually large and easily identifiable. The pAgNOR>1 count is most probably less sensitive to variation in section thickness and variations in staining technique than mean counts. Moreover this parameter shows an excellent reproducibility (Xie et al. 1997*a* and *b*).

The biological significance of nuclear organizer regions (NORs) remains unknown. Several studies have, however, demonstrated a relationship between AgNOR quantity and cell proliferation (Mirre and Knibiehler, 1982; Carbajo et al. 1993; Ruschoff et al. 1994). Usually, resting cells have only one AgNOR (Mirre and Knibiehler, 1982; Carbajo et al. 1993). The number of AgNORs increase from early $G_1$-phase to late S-/$G_2$-phases (Carbajo et al. 1993; Ruschoff et al. 1994). Further evidence for the idea that AgNOR counts represent a marker for proliferative activity has been presented in a study using double staining with Ki67 and AgNOR (Mourad et al. 1994). While Ki67-negative cells only had one to three, the Ki67-positive cells had 2–12 AgNORs. Similarly, both pAgNOR and mAgNOR counts have been found to correlate with other proliferative markers, such as the S-phase fraction, the BrdU-labelling index and Ki67 labelling index (Mourad et al. 1992, 1993, 1994). These findings suggest that pAgNOR>1 reflects some aspects of the tumour proliferative activity, as does the mean AgNOR score. Because of the simplicity of the pAgNOR>1 counting method and a higher degree of reproducibility, pAgNOR>1 rather than mAgNOR is, in our view, the preferred AgNOR parameter (Xie et al. 1997*a* and *b*).

Despite the fact that T-stage in glottic carcinomas correlates well with prognosis (Vermund et al. 1990), we found no association between Bax expression and T-stage. The reason for this somewhat contradictory finding may be that the Bax expression was not a statistically strong parameter and that the two parameters pick out different groups of patients. Neither did we find any correlation between Bax expression and pAgNOR>1 or mAgNOR scores. This is consistent with the results of a study on breast adenocarcinomas in which the S-phase fraction was used as a parameter reflecting the proliferative activity (Krajewski et al. 1995). When we combined Bax expression and pAgNOR>1 for treatment response (see Figures 5 and 6), a strong cooperative relationship between these two variables emerged that predicted 29 of the 33 failures and 34 of the 36 non-failures.

Our investigation confirms that AgNOR enumeration is a prognostic marker and further suggests that Bax expression is a potential prognostic marker. Whereas Bax expression may reflect the response to radiation-induced apoptosis, AgNOR counts appear to be a measure of some aspects of the proliferative activity. The combination of these two parameters emerged as a strong discriminator regarding treatment failures in non-metastatic glottic carcinomas. Thus, strategies combining assessment of tumour proliferation and apoposis-related proteins may prove to be fruitful in the search for new prognostic tumour markers in squamous cell carcinomas of the head and neck.

### ACKNOWLEDGEMENTS

This study was supported by the Norwegian Cancer Society, the Anders Jahre Foundation and Torstedt's Foundation for Cancer Research.

### REFERENCES

Bargou RC, Wagener C, Bommert K, Mapara MY, Daniel PT, Arnold W, Dietel M, Guski H, Feller A, Royer HD and Dorken B (1996) Overexpression of the death-promoting gene bax-alpha which is downregulated in breast cancer restores sensitivity to different apoptotic stimuli and reduces tumour growth in SCID mice. *J Clin Invest* **97**: 2651–2659

Bockmühl U, Bockmühl F, Dimmer V and Kunze K-D (1992) "Nucleolar Organizer Regions" (AgNORs) als Factor für die Prognose des Larynxkarzinoms? *Laryngo–Rhino–Otol* **71**: 137–141

Boise LH, Gottschalk AR, Quintans J and Thompson CB (1995) Bcl-2 and Bcl-2-related proteins in apoptosis regulation. *Curr Topics Microbiol Immunol* **200**: 107–121

Carbajo S, Orfao A, Vicente-Villardon JL and Carbajo-Perez E (1993) Expression of silver-stained nucleolar organizer regions is coupled to cell cycle in rat thymic cells. *Cytometry* **14**: 46–52

Chresta CM, Masters JRW and Hickman JA (1996) Hypersensitivity of human testicular tumours to etoposide-induced apoptosis is associated with functional p53 and a high Bax:Bcl-2 ratio. *Cancer Res* **56**: 1834–1841

Contractor H, Ruschoff J, Schulze-Seemann X and Ulshöfer B (1989) Prognostic significance of NOR analysis in prostatic cancer. *Urol Res* **17**: 327–332

Delahunt B, Bethwaite PB, Nacey JN and Ribas JL (1993) Proliferating cell nuclear antigen (PCNA) expression as a prognostic indicator for renal cell carcinoma: comparison with tumour grade, mitotic index, and silver-staining nucleolar organizer region numbers. *J Pathol* **170**: 471–477

Gazzaniga P, Gradilone A, Vercillo R, Gandini O, Silvestri I, Napolitano M, Albonici L, Vincenzoni A, Gallucci M, Frati L and Agliano AM (1996) Bcl-2/Bax mRNA expression ratio as prognostic factor in low-grade urinary bladder cancer. *Int J Cancer* **69**: 100–104

Hirsh SM, DuCanto J, Caldarelli DD, Hutchinson JC and Coon JS (1992) Nucleolar organizer regions in squamous cell carcinoma of the head and neck. *Laryngoscope* **102**: 39–44

Kitada S, Krajewski S, Miyashita T, Krajewska M and Reed JC (1996) γ-Radiation induces upregulation of Bax protein and apoptosis in radiosensitive cells in vivo. *Oncogene* **12**: 187–192

Kolar Z, Zabransky T, Mattler K and Zabransky E (1992) Argyrophilic nucleolar organizer regions in breast cancer: prognostic significance. *Cesk Patol* **28**: 193–200

Krajewski S, Blomqvist C, Franssila K, Krajewska M, Wasenius VM, Niskanen E, Nordling S and Reed JC (1995) Reduced expression of proapoptotic gene Bax is associated with poor response rates to combination chemotherapy and shorter survival in women with metastatic breast adenocarcinoma. *Cancer Res* **55**: 4471–4478

Mirre C and Knibiehler B (1982) A re-evaluation of the relationships between the fibrillar centres and the nucleolus-organizing regions in reticulated nucleoli: ultrastructural organization, number and distribution of the fibrillar centres in the nucleolus of the mouse Sertoli cell. *J Cell Sci* **55**: 247–259

Mourad WA, Erkman-Balis B, Livingston S, Shoukri M, Cox CE, Nicosia SV and Rowlands DT Jr (1992) Argyrophilic nucleolar organizer regions in breast carcinoma: correlation with DNA flow cytometry, histopathology, and lymph node status. *Cancer* **69**: 1739–1744

Mourad WA, Connelly JH, Sembera DL, Atkinson EN and Bruner JM (1993) The correlation of two argyrophilic nucleolar organizer region counting methods with bromodeoxyuridine-labelling index: a study of metastatic tumours of the brain. *Hum Pathol* **24**: 206–210

Mourad WA, Sneige N, Katz RL and Ordonez NG (1994) Correlation of two AgNOR counts with Ki-67 labelling index: a study in fine-needle aspirates of lymphoproliferative disorders and breast carcinoma. *Diagn Cytopathol* **10**: 113–119

Oltvai ZN, Milliman CL and Korsmeyer SJ (1993) Bcl-2 heterodimerizes in vivo with a conserved homolog, Bax, that accelerates programmed cell death. *Cell* **74**: 609–619

Pich A, Pisani P, Kzengli M, Cappello N and Navone R (1991) Argyrophilic nucleolar organizer region counts and prognosis in pharyngeal carcinoma. *Br J Cancer* **64**: 327–332

© Cancer Research Campaign 1998

Piffkò J. Bànkfalvi A˙. Öfner D. Bryne M. Rasch D. Joos U. Böcker W and Schmid KW (1997) Prognostic value of histobiological factors (malignancy grading and AgNOR content) assessed at the invasive tumour front of oral squamous cell carcinomas. *Br J Cancer* **75**: 1543–1546

Ploton D. Menager M. Jeannesson P. Himber G. Pigeon F and Adnet JJ (1986) Improvement in the staining and in the visualization of the argyrophilic proteins of the nucleolar organizer region at the optical level. *Histochem J* **18**: 5–14

Reed JC (1994) Bcl-2 and the regulation of programmed cell death. *J Cell Biol* **124**: 1–6

Ruschoff J. Bittinger A. Neumann K and Schmitz-Moormann P (1990) Prognostic significance of nucleolar organizer regions (NORs) in carcinomas of the sigmoid colon and rectum. *Pathol Res Pract* **186**: 85–91

Ruschoff J. Fauser G. Knuchel R and Hofstadter F (1994) AgNOR quantification with special reference to staining patterns. *Zentralbl Pathol* **140**: 23–30

Sakakura C. Sweeney EA. Shirahama T. Igarashi Y. Hakomori S. Nakatani H. Tsujimoto H. Imanishi T. Ohgaki M. Ohyama T. Yamazaki J. Hagiwara A. Yamaguchi T. Sawai K and Takahashi T (1996) Overexpression of bax sensitizes human breast cancer MCF-7 cells to radiation-induced apoptosis. *Int J Cancer* **67**: 101–105

Sano K. Takahashi H. Fujita S. Inokuchi T. Pe MB. Okabe H and Tsuda N (1991) Prognostic implication of silver-binding nucleolar organizer regions (AgNORs) in oral squamous cell carcinoma. *J Oral Pathol Med* **20**: 53–56

Searle J. Collins DJ. Harmon B and Kerr JFR (1973) The spontaneous occurrence of apoptosis in squamous carcinomas of the uterine cervix. *Pathology* **5**: 163–169

Stoetzer OJ. Nussler V. Darsow M. Gullis E. Pelka-Fleischer R. Scheel U and Wilmanns W (1996) Association of bcl-2. bax. bcl-xL and interleukin-1 beta-converting enzyme expression with initial response to chemotherapy in acute myeloid leukemia. *Leukemia* **10** (suppl. 3): 18–22

Thomas A. El Rouby S. Reed JC. Krajewski S. Silber R. Potmesil M and Newcomb EW (1996) Drug-induced apoptosis in B-cell chronic lymphocytic leukemia: relationship between p53 gene mutation and bcl-2/bax proteins in drug resistance. *Oncogene* **12**: 1055–1062

Vermund H. Boysen M. Brandenburg JH. Evensen J. Jacobsen A-B. Kaalhus O. Tausjö J. Thorud E. Wiley AL and Winther F (1990) Primary irradiation. surgery or combined therapy in squamous cell carcinoma of the larynx. A comparison of treatment results from two centres. *Acta Oncol* **29**: 489–503

Wagener C. Bargou RC. Daniel PT. Bommert K. Mapara MY. Royer HD and Dorken B (1996) Induction of the death-promoting gene bax-alpha sensitizes cultured breast-cancer cells to drug-induced apoptosis. *Int J Cancer* **67**: 138–141

Wyllie AH (1985) The biology of cell death in tumours. *Anticancer Res* **5**: 131–136

Xie X. Stenersen TC. Clausen OPF and Boysen M (1997*a*) Nucleolar organizer regions and prognosis in glottic squamous cell carcinoma. *Head Neck* **19**: 20–26

Xie X. Clausen OPF. Sudbö J and Boysen M (1997*b*) Diagnostic and prognostic value of nuclear organizer regions in normal epithelium. dysplasia and squamous cell carcinoma of the oral cavity. *Cancer* **79**: 2200–2008

Öfner D. Tötsch M. Sandbichler P. Hallbrucker C. Margreiter R. Mikuz G and Schmid KW (1990) Silver stained nucleolar organizer region proteins (AgNORs) as a predictor of prognosis in colonic cancer. *J Pathol* **162**: 43–49

© Cancer Research Campaign 1998